# An unusual case of a well-differentiated neuroendocrine tumour of the ileum with peritoneal carcinomatosis: a case report

**DOI:** 10.1186/s12957-015-0585-7

**Published:** 2015-05-02

**Authors:** Andrea Celotti, Giuseppe Pulcini, Mattia Schieppati, Silvia Ministrini, Alfredo Berruti, Maurizio Ronconi

**Affiliations:** Surgical Clinic of Brescia, Piazzale Spedali Civili, 1, Brescia, Italy; Surgical Unit, Hospital of Gardone Val Trompia, Gardone Val Trompia, Italy; Oncologic Unit of Brescia, Brescia, Italy

**Keywords:** NET, Neuroendocrine tumours, Neuroendocrine carcinoma, NETs classification, Peritoneal carcinomatosis

## Abstract

Neuroendocrine tumours (NETs) are a family of neoplasms that come from neuroendocrine cells and express neural markers, such as synaptophysin or chromogranin A.

The current classifications of these tumours are presented by the WHO 2000 classification, based on histological parameters, and the WHO 2010 classification, based on the proliferative index, that divides the NETs into a neuroendocrine tumour of a low grade, neuroendocrine tumour of a intermediate grade and neuroendocrine carcinoma (NEC) of a high grade.

We are reporting a very rare case of a G1 low-grade neuroendocrine tumour (NET) of the ileum with a peritoneal carcinomatosis.

This case is challenging because the tumour expresses low proliferative index as G1 tumours, but it has an aggressive clinical behaviour such as node metastasis and peritoneal carcinomatosis.

The peritoneal carcinomatosis is not actually considered by the current classifications of NETs, so it is difficult to predict the prognosis of this patient.

## Background

This article represents the original observation of a low-grade neuroendocrine tumour of the ileum debuting with a bowel obstruction and peritoneal carcinomatosis.

Neuroendocrine tumours (NETs) are a family of neoplasms that arise from neuroendocrine cells and express neural markers, such as synaptophysin or chromogranin A. They are mainly distributed in the gastrointestinal tract. Pancreatic neuroendocrine tumours are included in this category (gastrointestinal-pancreatic neuroendocrine tumours (GPNETs). The behaviour of NETs differs by anatomic site [1], so it is important to define the tumour localization for prognostic stratification.

Current classifications of NETs are the 2000 WHO and the 2010 WHO classifications. The first is based on histological parameters such as size, depth of invasion, angiolymphatic invasion and metastases, and it divides the tumours into well-differentiated neuroendocrine tumour (WDNT), well-differentiated neuroendocrine carcinoma (WDNC) and poorly differentiated neuroendocrine carcinoma (PDNC). The 2010 WHO classification, instead, considers the proliferative activity of the tumours on the basis of their expression of the Ki-67 index or the mitotic count [1-10], and it divides the tumours into neuroendocrine tumour (NET) of low grade, neuroendocrine tumour of intermediate grade and neuroendocrine carcinoma (NEC - high grade). So the two classifications consider different aspects of these tumours and use different nomenclature.

Well/moderately differentiated NETs are relatively low-aggressive tumours, with a rather indolent disease course and good prognosis in most patients. The lymph nodes and the liver are the prevalent metastatic sites. Peritoneal carcinomatosis is a frequent complication of high-grade aggressive tumours, but to our knowledge, it has never been described in patients with low-grade NETs.

The aim of our work is to report the case of low-grade NET of the ileum with peritoneal carcinomatosis at the first diagnosis of the disease.

## Case presentation

We describe the case of a 64-year-old man admitted to our hospital for an intestinal obstruction caused by a tumour of the ileum associated with pelvic peritoneal carcinomatosis.

A preoperative CT scan showed an ileal solid mass of 4 cm in diameter with peritoneal fluid in Douglas and radiologic signs of intestinal obstruction. No liver nor thoracic focal lesions were found.

We performed an urgent surgical operation. At the opening of the peritoneal cavity, we found a stenotic ileal tumour, about 40 cm from the ileocecal valve, with a mesenteric adenopathy of 4 cm and pelvic peritoneal carcinomatosis. Ileal tract of 30 cm was resected and a manual L-L anastomosis performed.

**Figure 1:**
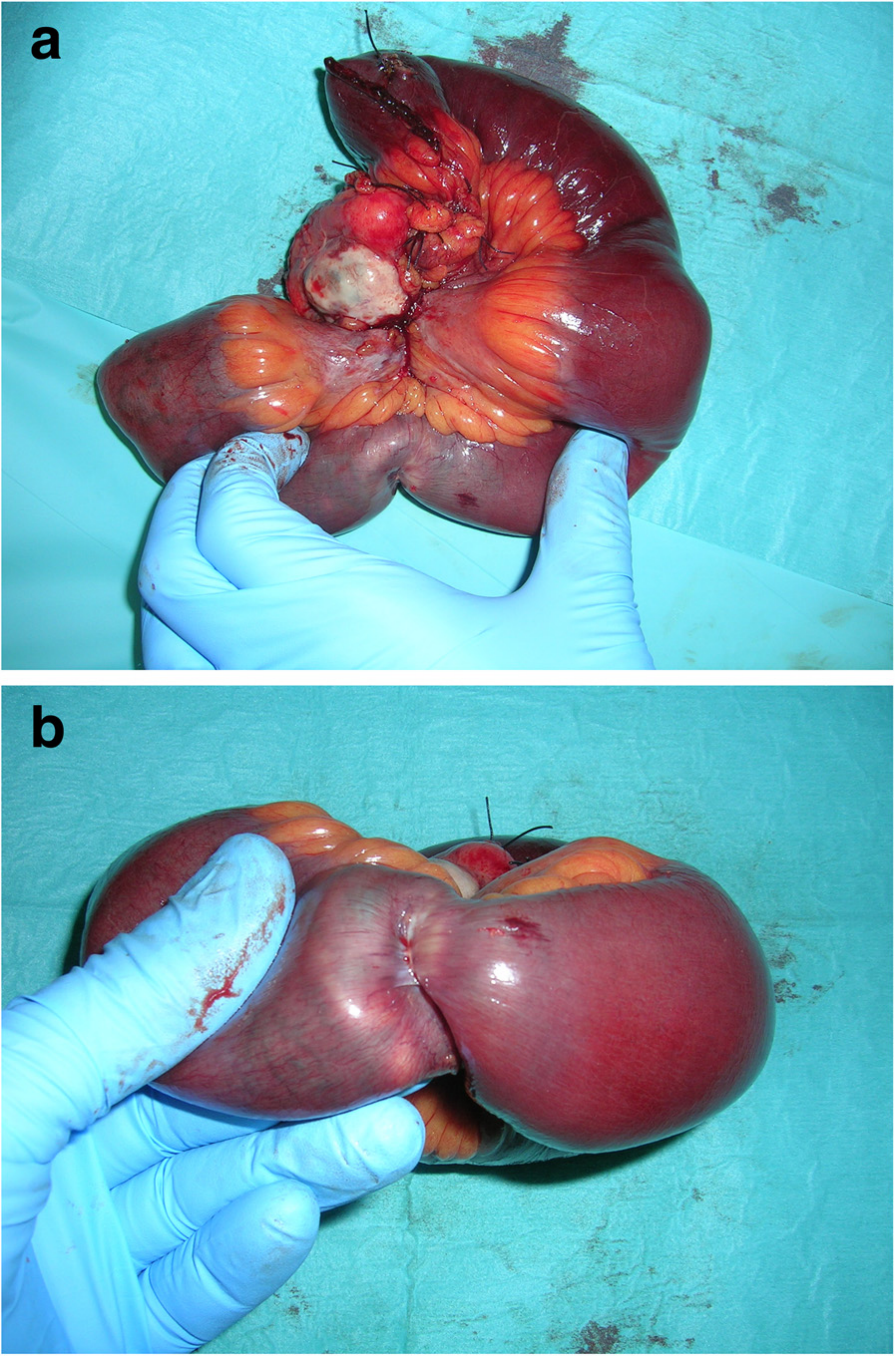
**a**, **b** Macroscopic aspects of ileal primitive tumour.

Pathologic evaluation of the resected specimen confirmed the diagnosis of a neuroendocrine tumour 2.7 × 2 cm in size, with a metastatic node mass of 4 cm in diameter. Only one node was positive for cancer localization. The tumour presented serosa and perivisceral fat infiltrations, perineural and lymphatic vessels invasion and mesenteric implants (Figure 1a, b). Our diagnosis was pT4N1 according to the 2010 American Joint Committee on Cancer (AJCC) classification (Table 1).

**Table 1:** **2000 TNM NETs classification**

**Primary tumour (T)**		
T1	Tumour invades lamina propria or submucosa and is size 1 cm or less	
T2	Tumour invades muscolaris propria or is size >1 cm	
T3	Tumour invades through the muscolaris propria into the subserosa or into the nonperitonealized tissue	
T4	Tumour invades the visceral peritoneum (serosa) or any other organs or structures	
Regional lymph nodes (N)		
N0	No regional lymph nodes metastasis	
N1	Regional lymph nodes metastasis	
Distant metastasis (M)		
M0	No distant metastasis	
M1	Distant metastasis	
Stage		
I	T1N0M0	
IIA	T2N0M0	
IIB	T3N0M0	
IIIA	T4N0M0	
IIIB	AnyT N1M0	
IV	AnyT anyN M1	
Differentiation/grade	Mitotic Count (10 HPF)	Ki-67 index (%)
Well differentiated		
Low-grade	<2	> or = 2
Intermediate-grade	2 to 20	3 to 20
Poorly indifferentiated	>20	>20

An immunohistochemistry analysis revealed synaptophysin and chromogranin A positivity and a Ki-67 expression <1%. Mitotic count/10 was 2 × 10 high-power fields (HPF) and cells showed well differentiation. So, according to the novel WHO 2010 classification for GPNETs [1,4-6,11], we classified this tumour as G1-NET.

**Figure 2:**
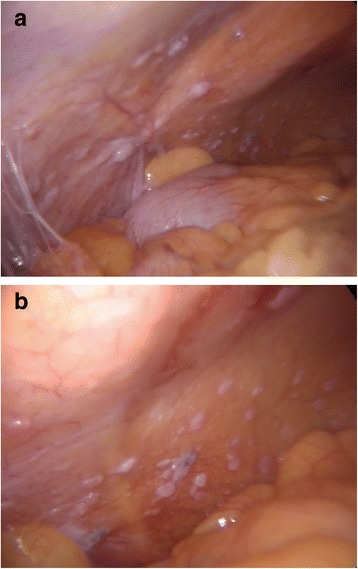
**a**, **b** Laparoscopic second look.

The case was discussed in a multidisciplinary team, consisting of oncologists, surgeons, radiation oncologists and radiologists. To our knowledge, this is the first case reported in literature of a low-grade NET with concomitant peritoneal carcinomatosis. To exclude the presence of another primitive tumour, we performed a positron emission tomography with gallium-labeled somatostatine analogues (DOTA-NOC PET) and a second look in a laparoscopy way (Figure 2a, b), with the aim to achieve a complete systemic and peritoneal staging [12-14].

**Figure 3:**
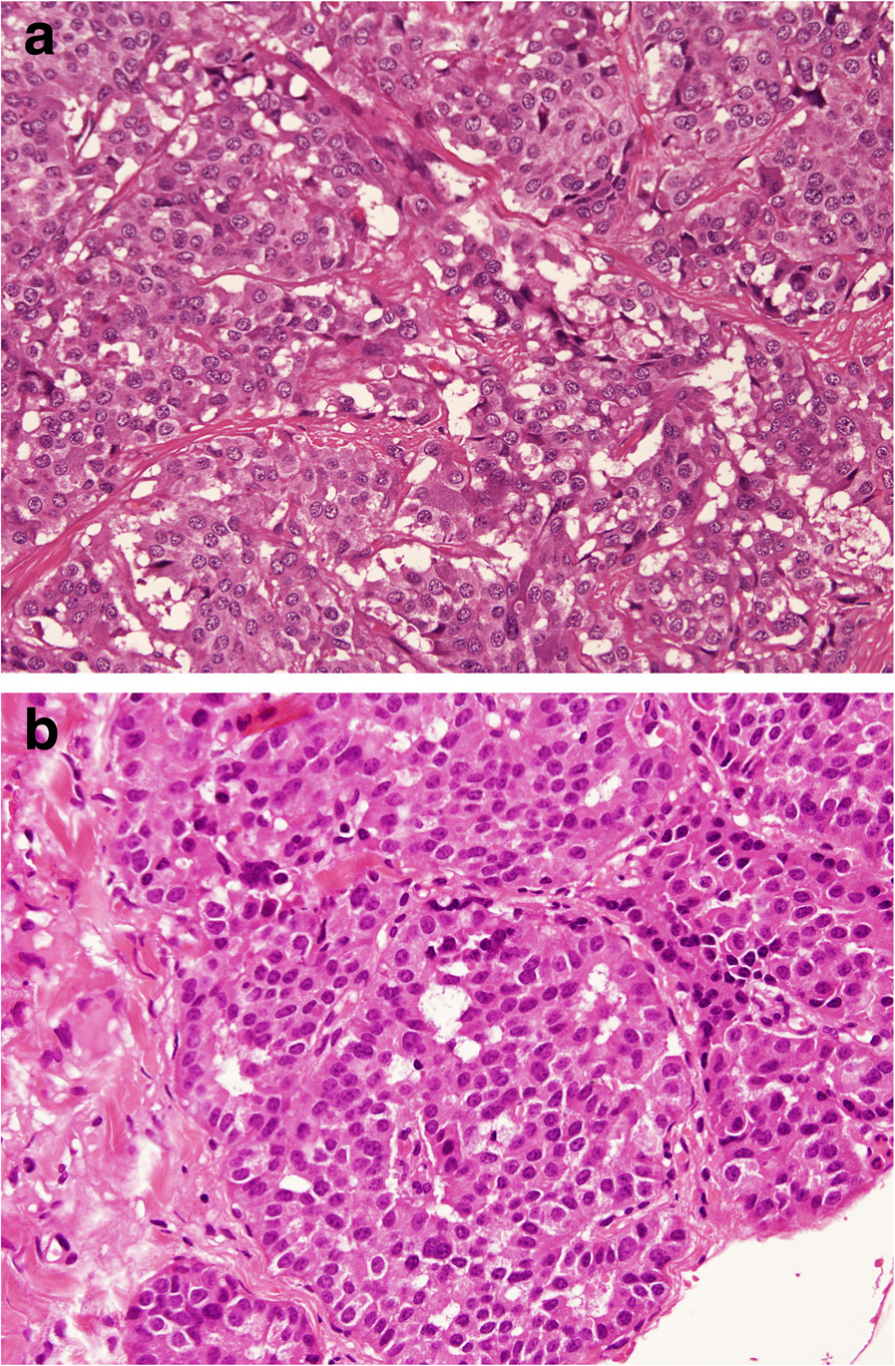
Microscopic and molecular aspects of the ileal tumour **(a)** and the carcinomatosis nodules **(b)**.

**Figure 4:**
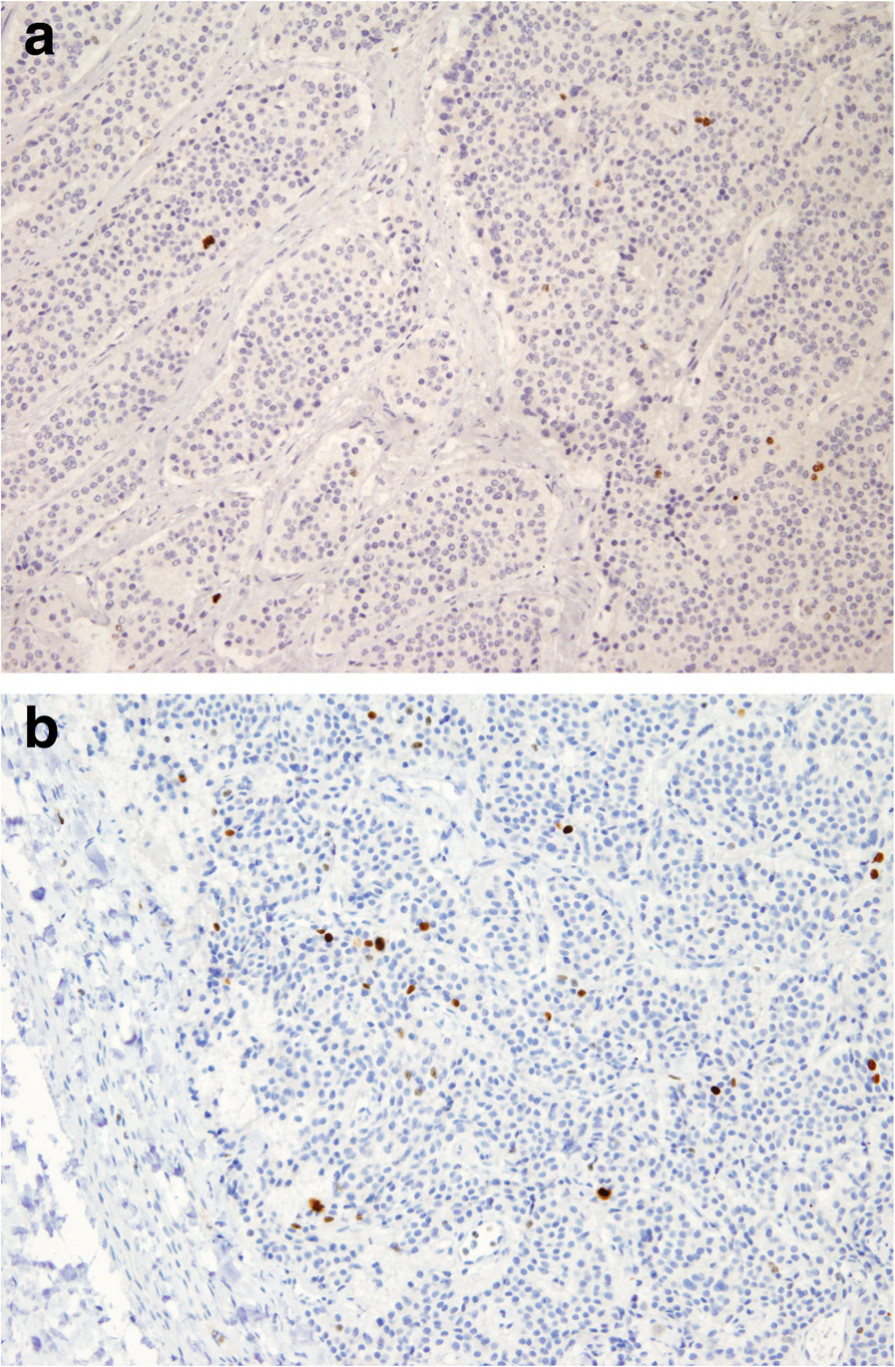
Microscopic and molecular aspects of the ileal tumour **(a)** and the carcinomatosis nodules **(b)**.

**Figure 5:**
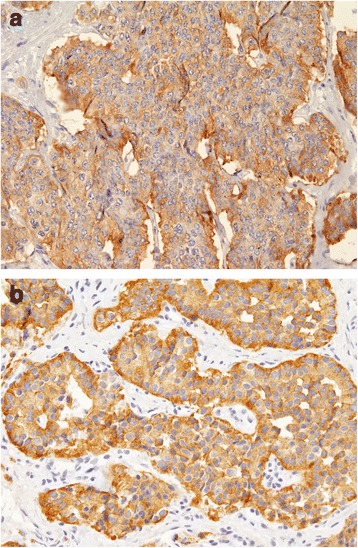
Microscopic and molecular aspects of the ileal tumour **(a)** and the carcinomatosis nodules **(b)**.

During the second surgical operation, multiple biopsies of the metastatic nodules and parietal peritoneum were done (left and right pelvic peritoneum, left and right diaphragmatic peritoneum) and a peritoneal cytology was performed. The exploration revealed no other tumours. According to the classification of peritoneal carcinomatosis, the laparoscopic staging showed a peritonial cancer index (PCI) of 4 [12-14]. Peritoneal cytology was negative for malignancy. At the histological examination, carcinomatosis nodules showed the same features of the primary tumour. So the final diagnosis was ileal G1 NET with peritoneal carcinomatosis T4N1M1, stage IV (Figures 3a, b, 4a, b, and 5a, b).

The patient is currently under treatment with somatostatine analogue and in follow-up with biannual DOTA-NOC PET; at present, he is disease free.

### Discussion

The gastropancreatic neuroendocrine neoplasms (GEP-NEN) are a heterogeneous group of tumours characterized by the expression of the neural antigens such as chromogranin A or synaptophysin.

They are relatively rare but with an increasing incidence rate in the last years [2].

The term *carcinoid* was proposed for the first time in 1907 by S. Oberndorfer, and it is nowadays traditionally used although it is not mentioned in the current classification of the neuroendocrine neoplasms (NEN).

In 2000, the WHO revised the previous classification system of 1980. Neuroendocrine tumours were divided into three groups, considering various histological parameters, such as size, depth of invasion, angiolymphatic invasion and metastases: WDNT or carcinoid; WDNC or malignant carcinoid; PDNC [1,3].

In 2010, a revised version of the previous WHO classification appeared (Table 2), not based on histological parameters, but on proliferation rate, expression of Ki-67 index, or on mitotic count, calculated by the number of mitosis/10 HPF [4-6].

**Table 2:** **2010 WHO NETs classification**

**Definition**	**Grade**	**Ki-67%**	**Mitotic index**
NET - neuroendocrine tumour, low grade	G1	≤2	<2
NET - neuroendocrine tumour, intermediate grade	G2	3 to 20	2 to 20
NET - neuroendocrine carcinoma, high grade	G3	>20	>20

The category of well-differentiated neuroendocrine carcinoma of WHO 2000 does not exist anymore in this new classification, and all tumours are evaluated malignant with the potential to metastasize [1].

In a recent study of Yamaguchi T. et al. [7], considering a group of 45 patients with diagnosis of G1 and G2-NET, metastases were observed also in the G1-NET. In the metastatic group of seven patients, four showed a Ki-67 ≤ 2. Metastasis was observed in the liver, lungs and lymph nodes, and one patient presented a local recurrence.

Clinical behaviour of the NEN is very heterogeneous: NEC are considered extremely aggressive, with a bad prognosis, instead of NET-G1, that are relatively indolent [6,15].

Miller HC et al. analyzed the metastatic rate in a cohort of 161 patients with NEN and found out that metastasized tumours were noted in 46.1% of the G1 cases, 77.8% of the G2 cases and 100% of the G3 cases. In spite of this, relatively high incidence of metastasis also in G1 tumour; 87% of G1 cases are alive after 3 years [16].

So it is mandatory in the pathology report to specify the grade or differentiation of the tumour. Indeed, the terms neuroendocrine carcinoma and neuroendocrine tumour, without reference to these parameters, are considered inadequate for prognosis or therapy [6,8].

The proliferative rate can be assessed as the number of mitosis per unit area of tumour or as the percentage of proliferation marker Ki-67.

Despite the new classification of NET, WHO 2010, many works are based on the old WHO 2000 classification, making an analytic comparison of the data very difficult.

Some studies that compared the histological features of the WHO 2000 classification to proliferative index of the WHO 2010 classification in predicting metastasis have shown that the Ki-67 index is the best parameter [4,7]. Metastasis is more frequent in the liver, lymph nodes, lungs, bones and other organs. A rare case of a solitary atrial myocardial metastasis is reported [17]. Liver metastasis were found between 20% and 60% of the NETs [18,19].

In literature, some cases of small bowel NETs-peritoneal carcinomatosis are reported [12,13,19-22], but at our knowledge, a case of well-differentiated neuroendocrine tumour of ileum with single node metastasis and peritoneal carcinomatosis has never been described.

Metastatic G1 well-differentiated NETs are described in literature [7,15,20,21,23,24], and the liver is the most frequent site of neoplastic spreading, but there are no reports about peritoneal carcinomatosis in G1 NET.

G1 NETs are considered relatively indolent [6]. Strosberg J et al. reported a survival rate at 2 and 5 years of 100% and 85%, respectively, for G1 NETs, and considering the time between diagnosis of metastasis and death from any cause, median survival was not reached in the well-differentiated NETs [20].

In spite of this benign behaviour of G1 NETs, we report a case of potentially aggressive well-differentiated G1 NET. Although the low proliferative index and mitotic rate (Ki-67 < 1% and mitotic count 2x10 HPF), the tumour showed lymphatic metastasis, serosa and perivisceral fat infiltrations, perineural and lymphatic vessels invasion, mesenteric implants and above all peritoneal carcinomatosis.

We decided to perform a second surgical operation for PCI staging in a laparoscopic way because some studies demonstrated that laparoscopy is a safe and accurate technique for peritoneal carcinomatosis staging [14,25].

**Figure 6:**
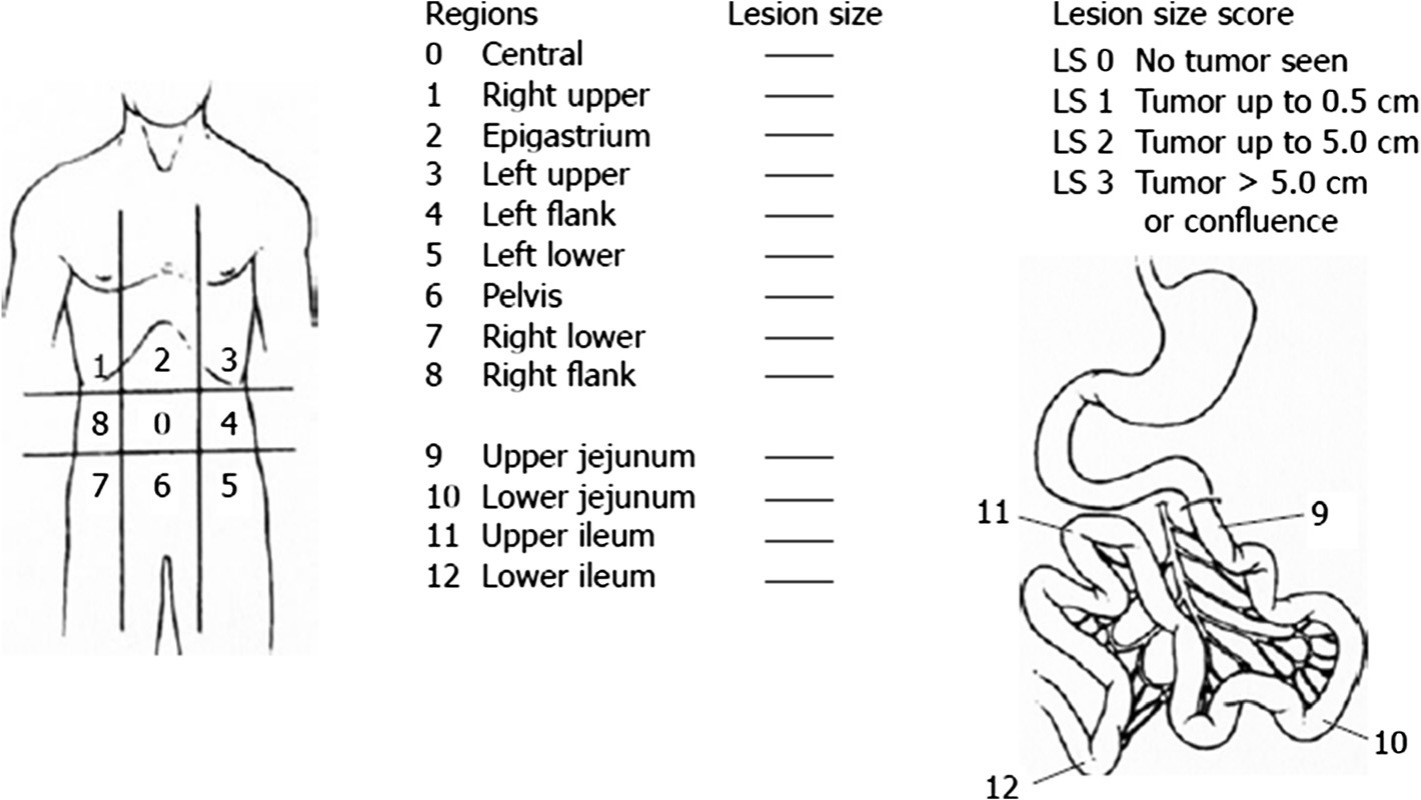
PCI scoring system.

We started with adhesiolysis procedure, and after, the peritoneal cavity was totally explored for the allocation of PCI using the Sugarbaker scoring system (Figure 6).

Such as for ovarian cancer, we accomplished a peritoneal washing for cytology examination and multiple random peritoneal biopsies of normal surfaces and neoplastic implants [25]. We think that, during surgery, a complete peritoneal cavity exploration should be always performed for these tumours to avoid a wrong staging of the disease, and laparoscopy could be, in selected cases, a useful technique.

Peritoneal nodes of carcinomatosis presented the same features of the primary tumour, and also Ki-67 and mitotic count were the same. According to AJCC 2010, tumour was classified as pT4N1, stage IV [8,10,11,26-29].

In some cases metastasis have a more aggressive behaviour in relation to primary tumours. A recent study compared the expression of the Ki-67 index between primary tumours and liver metastasis in 30 patients with NETs [23]. Actually, in one third of the patients with well-differentiated NETs, liver metastasis had an elevated Ki-67 index. Four cases were small bowel NETs and three of these were upgraded from G1 to G2 (two cases) or from G2 to G3 (one case).

In our, case both the ileal tumour and the carcinomatosis nodules showed the same Ki-67 index and a well differentiation grade. This means that the tumour has an unusual behaviour because it shows immunohistochemical markers of low malignancy, such as other indolent G1 NET, but histological features of high aggressiveness.

The work of Pape UF et al. [28], comparing the prognostic relevance of the TNM classification system to the grading system, reports a 5-year survival rate of 55.4% for stage IV NETs, according to the TNM classification system and of 95.7% for G1 NETs, according to the grading system.

In a recent study, the 5-year probability of survival of 458 patients with stage IV midgut NETs is reported around 70% against 100% of stage I, and the 5-year probability of survival of 499 patients with low-grade midgut NETs is near 100% [29].

The prognosis of the well-differentiated NET with peritoneal carcinomatosis could not be established since neither the WHO 2010 classification nor the TNM classification system could be useful for prognostic stratification.

## Conclusions

GPNETs are rare tumours, and large case series are difficult to collect and analyze, although their incidence is increasing. For these reasons, it is crucial for scientists to employ a universally accepted diagnostic system with a common language.

Both histological parameters (such as tumour dimensions, lymphatic and vascular invasion, nodes involvement, metastasis, etc.) and proliferative index (such as Ki-67 or mitotic count) are important in prognostic stratification.

Our case is challenging because the tumour expresses low proliferative index such as G1 tumours, but it has an aggressive clinical behaviour such as node metastasis and peritoneal carcinomatosis.

The peritoneal carcinomatosis is actually not considered by the current classifications of NETs, so it is difficult to predict the prognosis of this patient.

## Abbreviations

NET: neuroendocrine tumour
NEC: neuroendocrine carcinoma
WDNT: well-differentiated neuroendocrine tumour
WDNC: well-differentiated neuroendocrine carcinoma
PDNC: poorly differentiated neuroendocrine carcinoma
GPNETs: gastrointestinal-pancreatic neuroendocrine tumours
GEP-NEN: gastropancreatic neuroendocrine neoplasms
